# Guided evolution of *in silico *microbial populations in complex environments accelerates evolutionary rates through a step-wise adaptation

**DOI:** 10.1186/1471-2105-13-S10-S10

**Published:** 2012-06-25

**Authors:** Vadim Mozhayskiy, Ilias Tagkopoulos

**Affiliations:** 1 Department of Computer Science and UC Davis Genome Center, University of California Davis, Davis, California, 95616, USA

## Abstract

**Background:**

During their lifetime, microbes are exposed to environmental variations, each with its distinct spatio-temporal dynamics. Microbial communities display a remarkable degree of phenotypic plasticity, and highly-fit individuals emerge quite rapidly during microbial adaptation to novel environments. However, there exists a high variability when it comes to adaptation potential, and while adaptation occurs rapidly in certain environmental transitions, in others organisms struggle to adapt. Here, we investigate the hypothesis that the rate of evolution can both increase or decrease, depending on the similarity and complexity of the intermediate and final environments. Elucidating such dependencies paves the way towards controlling the rate and direction of evolution, which is of interest to industrial and medical applications.

**Results:**

Our results show that the rate of evolution can be accelerated by evolving cell populations in sequential combinations of environments that are increasingly more complex. To quantify environmental complexity, we evaluate various information-theoretic metrics, and we provide evidence that multivariate mutual information between environmental signals in a given environment correlates well with the rate of evolution in that environment, as measured in our simulations. We find that strong positive and negative correlations between the intermediate and final environments lead to the increase of evolutionary rates, when the environmental complexity increases. Horizontal Gene Transfer is shown to further augment this acceleration, under certain conditions. Interestingly, our simulations show that weak environmental correlations lead to deceleration of evolution, regardless of environmental complexity. Further analysis of network evolution provides a mechanistic explanation of this phenomenon, as exposing cells to intermediate environments can trap the population to local neighborhoods of sub-optimal fitness.

## Background

From microbes to vertebrates, organisms are constantly subjected to evolutionary processes that lead to adaptation and phenotypic variation. Whether evolutionary forces lead to new and rapidly evolving species, as it is in the case of adaptive radiation, or are responsible for phenotypic divergence within a species, the main underlying mechanism by which complex behavior arises remains the same: gradual accumulation of selected genetic mutations and epigenetic changes gives rise to a myriad of anatomical, physiological and behavioral expressions. Although the notion that evolution, niche adaptation, and phenotypic variation leads to "endless forms most beautiful" can be traced back to Darwin [1], it was only in the last decade that with the advent of high-throughput sequencing and profiling techniques, we were able to understand the mechanisms by which mutations give rise to novel traits. Remarkably, it has been shown that even single mutations, such as nucleotide polymorphisms, can yield phenotypes that are significantly dissimilar [2]. The same holds for the rewiring of the gene regulatory and biochemical networks, as they were found to exhibit a highly degree of evolvability [3,4], yet preserve phenotypic robustness when under stabilizing selection and in the presence of disrupting mutations [5,6].

A challenging task is to identify the environmental and organism-specific characteristics that allow the rapid adaptation from past to new environments. Recently, the theory of "Facilitated Variation" provided a unifying framework according to which, organisms are under selection to develop conserved core components that can be reused to quickly adapt to novel environments. Computer simulations with logic gates and RNA secondary structures have demonstrated that facilitated variation spontaneously emerges during evolution [7], while theoretical models provide support that varying environments can affect evolution [8] and can give rise to modular structures [9].

**Figure 1:**
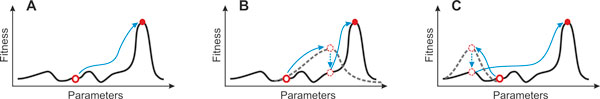
**Step-wise evolution**. Adaptation to a complex environment (**A**) can be accelerated (**B**) or decelerated (**C**) if guided through intermediate steps of a lesser complexity. Fitness profile for a population evolving in a target complex environment (solid black curve) is a multidimensional surface with multiple local maxima. Adaptation to intermediate environments (dashed grey fitness profiles) can direct evolution towards to (or away from) the global fitness maximum of the target environment.

Here, we posit that evolution can be both accelerated and decelerated through step-wise adaptation to novel environments. In complex environments, organisms have to explore a large parameter space before settling in stable fitness points (Figure 1A). Local minima and discontinuities may lead to sub-optimal fitness peaks, from where it may be difficult, or even infeasible, to escape. In addition, it has been shown that phenotypes that occupy flatter regions of the fitness surface are more robust to mutations, a phenomenon that was coined as "survival of the flattest"[10]. Intermediate environments can lead to fitness neighborhoods from where it is easier for an organism to adapt to novel environments (Figure 1B). However, exposure to an intermediate environment may also have the opposite effect, as it can result to neighborhoods of poor fitness potential upon transition from one environment to another (Figure 1C). To further investigate this hypothesis, we first define metrics to quantify environmental complexity, and then proceed to measure the rate of evolution in multi-scale simulations of evolving microbial populations under five dynamic environments.

## Methods

We used a multi-scale microbial simulator to perform simulations of microbial populations in five environments with distinct temporal dynamics. The multi-scale simulator employs abstract, multi-scale models of basic sub-cellular phenomena related to expression (transcription, translation, protein modification, degradation, etc.), evolution (mutation, gene duplication, gene deletion, etc.), network regulation and other evolutionary processes (natural selection). It has been used successfully in the past to generate hypotheses related to regulatory network evolution in nutrient-limited microbial communities [11], and we have recently extended to include Horizontal Gene Transfer events [12].

**Figure 2:**
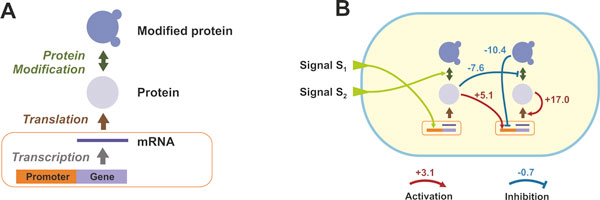
**Basic cellular model in our simulation framework**. (**A**) A "triplet": capturing the processes of transcription, translation, and post-translational modification. (**B**) Example of a gene regulatory and biochemical network in an organism where environmental signals (e.g. oxygen, temperature) regulate the expression of certain genes/proteins. The value at each node of the graph corresponds to the number of molecules of a given molecular species. Red/blue arrows denote positive/negative regulation and their corresponding weights.

In a simulation run, a population is composed of a fixed number of organisms. Each cell comprises of a number of "triplets" (three nodes): Gene/mRNA, Protein, and Modified Protein (Figure 2A). The Promoter/Gene/RNA node captures gene regulation and transcription, while the Protein and Modified Protein nodes capture translation and post-translational modification, respectively. Each organism has its own distinct gene regulatory and biochemical network (i.e. a collection of various triplets and weighted regulatory edges) that can be depicted as a directed weighted graph (see Figure 2B). The probability of molecule creation at each node and at each time step is a function of the regulatory effect of other nodes (activation or inhibitions) on that specific node, and the availability of substrate molecules. We model the molecule production probability as a two-level sigmoid function that captures a threshold and saturation effects for any given regulator and for the expression of any given node [11]. In addition to its regulatory network, each organism has a unique metabolic pathway which, when expressed, can metabolize available resources in the environment.

Mutational events (e.g. transcription rate changes, node duplications, node deletions, etc.) occur stochastically at any time point and on any node, thus changing its internal network and potentially its phenotype, which in this context is synonymous to the regulatory and metabolic pathway expression. The production and destruction of any molecule has an energy cost, as does the maintenance of molecular species (nodes). Organisms cannot directly sense the presence of resources; however they can potentially infer their future presence, if they are able to process information from various environmental signals through biochemical and regulatory interactions. Once an organism reaches a certain energy level, it undergoes division, increasing its genotype representation in the population, while its progeny replaces an existing organism so that the fixed size of the population is preserved (probability of an organism being replaced is inversely proportional to its energy level).

**Figure 3:**
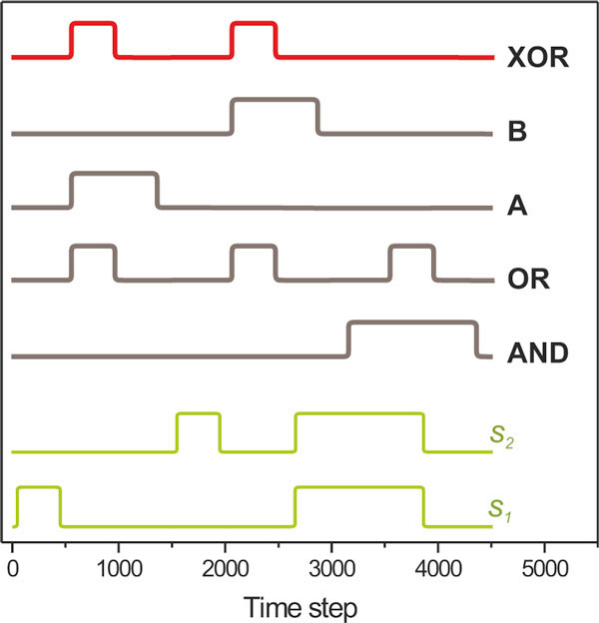
**Environments**. Environmental signals (green) and nutrient abundance for five environments (bottom to top: AND, OR, A, B, XOR) shown as a function of time steps within one epoch. Nutrient presence is a delayed function of the two signals. One epoch is shown for each environment, which consists of 4,500 time units.

For our simulations here, we used environments where two signals, *s_1 _*and *s_2_*, carry information regarding the presence of nutrients in the environment (Figure 3). The I/O characteristic of environments A and B is given by the logic *Nutrients Presence [A] = Delayed *(*s_1 _*AND NOT(*s_2_*)) and *Nutrients Presence [B] = Delayed *(NOT(*s_1_*) AND *s_2_*), respectively. This logic produces a single peak when *s_1 _*and *s_2 _*have the temporal characteristics of the waveform presented in Figure 3. Environments that encode an AND, OR and XOR gate where also used. The latter is also the environment with the most complex correlation structure, due to the fact that the XOR gate is not linearly separable. In addition, we introduced a delay in the signal/nutrient correlation (through the *Delayed() *function, which imposes a fixed delay of 500 time steps) to further increase the evolutionary complexity of the environment, as organisms now have to account for it through the topology and dynamics of the respective underlying networks. Similar observations were obtained with the absence of delay, although evolution was faster and resulted in simpler regulatory networks.

To assess the fitness level of each organism, we report the Pearson correlation between nutrient abundance and response protein expression level over a predefined interval of time, which we call an "epoch" (4,500 time units in our simulations). We stress that this similarity measure is used for visualization purposes as a proxy to each organism's fitness, and at no point participates or interferes with the selection or evolutionary trajectory of cells during the simulation. High correlation between nutrients and response protein concentration implies an efficient underlying mechanism to metabolize nutrients, as activation of this costly pathway takes place only when it confers an advantage to the organism.

## Results

### Environmental complexity and the rate of evolution

It is expected that the time it takes until a fit phenotype emerges is inversely proportional to the complexity of the environment. For instance, the gene regulatory and biochemical network of an organism which has to evolve a simple inverse relationship between signals and nutrients (i.e. the signal should be absent, for an action to take place), will be much less complex than the network of an organism which must capture an oscillatory dependency between inputs and outputs. The same is true for the evolution of networks in other contexts (social, financial, etc.) where inference and decision-making are an integral part of the network function. It is an open question, however, how we can quantify environmental complexity in a way that it captures the expected rate of evolution, or the time it will take for a fit phenotype to emerge.

**Figure 4:**
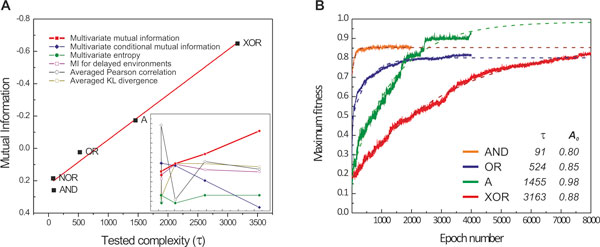
**Measuring environmental complexity by using multivariate mutual information**. (**A**) Multivariate mutual information between environmental signals and nutrient presence was found to correlate with the evolutionary rate, in experiments with 1024 cells, 5 environments, 10^7 ^time units, and 64 experiments per environment (replicates). Insert shows the lack of correlation of the evolutionary rate with other information measures (as reported in Table 1). (**B**) Fitness trajectories for AND, OR, A, and XOR environments averaged over 64 experiments; evolutionary rate is defined as the time constant *τ *of the exponential fit (*w*=*A_0_*-α*exp(-*t*/τ)) on the averaged fitness trajectory of the evolved populations (dashed curves and inset table).

To address this question, we evolved initially random populations in the five environments that we mentioned before, and then measured the rate of evolution over 32 simulation runs. Then, we considered several information-theoretic measures that capture the correlation between the observable inputs and latent output, and assessed their potential to predict the evolutionary rate (Table 1). We found that multivariate mutual information between environmental inputs (signals) and outputs (nutrients) correlates well with the rate of evolution for the environments that we studied (Figure 4A). As expected, the rate of evolution (measured as the time constant in an exponential fit) was slower for the case of XOR than any other environment tested.

**Table 1:** Information metrics of environmental complexity.

	Emergence of the organism with fitness *w*			
	*w *> 0.75		***w ***>**0.85**	
	Average speed, *epochs*	Success Rate	Average speed, *epochs*	Success Rate
Un-evolved → *AND*	39	64/64	236	64/64
Un-evolved → *OR*	1,179	57/64	> 4,000	25/64
Un-evolved → *A*	1,667	60/64	2,425	59/64
Un-evolved → *XOR*	5,241	26/64	9,583	21/64

*AND *→ *XOR*	4,891	7/16	> 6,000	7/16
*XOR *→ *AND*	250	14/16	1230	6/16

*AND *→ *OR*	> 4,000	9/16	--	2/16
*OR *→ *AND*	17	16/16	199	14/16

*A *→ *AND*	125	15/16	696	10/16
*AND *→ *A*	1477	14/16	2362	14/16

*A *→ *OR*	220	11/16	2200	10/16
*OR *→ *A*	56	16/16	56	16/16

*OR *→ *XOR*	210	15/16	2093	10/16
*XOR *→ *OR*	42	16/16	119	16/16

*A *→ XOR	240	14/16	887	11/16
{*A *&*B*} → *XOR ***+ HGT**	138	16/16	423	12/16
*XOR *→ *A*	10	16/16	17	16/16

A populations is considered to be evolved once its fitness is above *w *= 0.75; additional phenotype refinement is required to pass the *w *= 0.85 threshold. Success rate is the ratio of simulations with fitness above threshold after 4,000 epochs over the total number of simulations.

**Figure 5:**
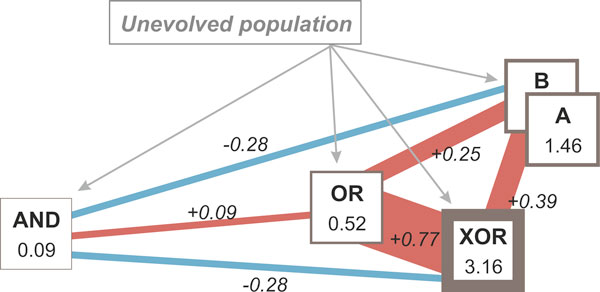
**Relative complexity and similarity of AND, OR, A, B, and XOR environments as a graph**. Node values correspond to the environmental complexity (squares, border thickness is proportional to the complexity). Pair-wise similarity is calculated as the Pearson correlation of nutrient presence between environments and corresponds to the edge value (red and blue represent correlation and anti-correlation, respectively). The environment of highest complexity, XOR, is part of a cluster of strongly correlated environments (A, B, OR, and XOR). Environment AND is the simplest one, and it is isolated from other environments (low or negative correlation).

Next, we calculated the environmental similarity between any two environments by measuring the pair-wise Pearson correlation of nutrient presence. Inclusion of the two signals would not alter the measured correlation, as the temporal dynamics of the two signals are the same in all environments that we consider. Figure 5 depicts the final "environmental network" that reflects the two measured quantities, the environmental quantity and similarity. We continued by testing whether evolution can be accelerated or decelerated by using different paths within the environmental network.

### Step-wise evolution through adaptation to environments of increased complexity

**Figure 6:**
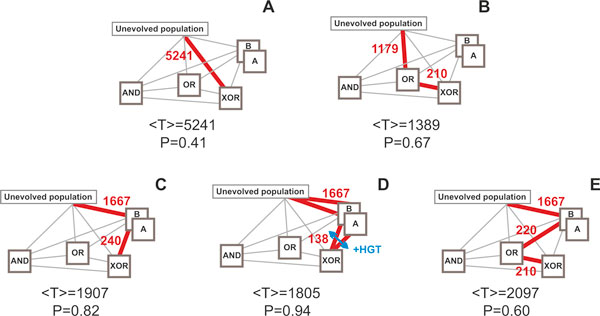
**Single-step and multi-step evolution towards an XOR phenotype**. The highlighted edges of each network correspond to the environmental transitions made during evolution (evolutionary path). The value of each edge corresponds to the average time to evolve a fit phenotype, in number of epochs. <T> and P are the average total time (traversing through the highlighted path) and the success probability (ratio of successful experiments over total experiments). **(A) **Direct evolution in an XOR environment is very slow and has a low probability of success **(B-C) **evolution to environments of lower complexity and subsequent evolution to the final environment accelerate evolution and boosts the number of successful experiments, **(D) **The presence of HGT in a population that pre-evolved in two simple environments further increase the evolution rate, **(E) **step-wise acceleration can occur in more than two steps, although at a lesser degree.

**Figure 7:**
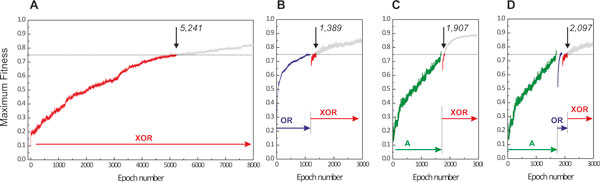
**Evolutionary trajectories for single-step and multi-step adaptation**. Vertical arrows show points where the average fitness in the population reaches the 0.75 threshold in a XOR environment. The 0.75 threshold guaranties that close to 99% of the population exhibits the I/O characteristics of the corresponding logical gate. **(A) **The evolutionary trajectory during single-step evolution in an XOR environment (averaged over 64 simulations), where it takes more than 5000 epochs to achieve the desired fitness; **(B-D) **If cells are first evolved in an OR and then in an XOR environment (guided, step-wise evolution), a fit phenotype emerges much faster (1,389 vs. 5,241 in the single-step evolution). Fitness discontinuity during transitions is due to the different correlations between the nutrients and the signals in the various environments. Similarity between environments (e.g. OR and XOR) result in cells that are exposed in the latter environments to already have certain non-zero fitness.

Interestingly, pre-evolving organisms to intermediate environments that have both lower complexity and strong, positive correlation to the final (target) environment, leads to a higher rate of evolution. We found that this effect was even more profound when positive correlation exists between the intermediate and final environments and in the presence of Horizontal Gene Transfer (HGT) [13]. As shown in Figure 6A, evolution in an XOR environment is usually slow and has low probability of success: 5241 avg. epochs, 26/64 successful simulations. Average *adaptation speed *or *time to a solution *is measured as an average number of epochs required for a population to reach a 0.75 fitness threshold in a given environment. However, when cells evolve in an OR environment first, and then transferred in an XOR environment, the average time to a solution decreases by a remarkable 73.5% and the success rate increases considerably (1389 avg. epochs, 67% success rate, Figure 6B). Similarly, if cells evolve in either A or B environment and then in an XOR environment the time is reduced by 63.5% to 1907 epochs (Figure 6C). This reduction is larger when the initial cell population evolves on parallel in A and B environments, and then a mixed population evolves in an XOR environment in the presence of HGT (1805 avg. epochs, Figure 6D). HGT allows the transfer of genetic material between cells of different lineages, rendering possible the transfer of gene clusters that confer a positive advantage to the target cell. This is particularly noticeable in mixtures of populations which have evolved in environments that are complementary to each other (such as A and B) and similar to the target environment (here XOR). Acceleration is also present in the case of paths with more than one intermediate goal (Figure 6E), although we expect that the overhead of exploring intermediate goals might eventually offset any acceleration gain present. Figure 7 illustrates the evolutionary trajectories in one-step and multi-step adaptation for the combinations that we examined; detailed statistics is shown in Table 2.

**Table 2:** Information metrics of environmental complexity.

Metric	*Adjusted R^2^*
**MI **(*S_1_*; *S_2_*; *N_shifted_*)	0.984
**MI **(*S_1_*; *S_2_*|*N_shifted_*)	0.981
**H **(*S_1_*; *S_2_*; *N_shifted_*)	0.028
**MI **(*S_1_*; *S_2_*; *N*)	-0.249
[**Corr**. (*S_1_*, *N_shifted_*) + **Corr**. (*S_1_*, *N_shifted_*)]/2	-0.332
[**K-L Div**. (*S_1_*||*N_shifted_*) + **K-L Div**. (*S_1_*||*N_shifted_*)]/2	-0.106

We evaluated the following measures on their correlation with the rate of evolution measured through experimental runs. From top to bottom: Multivariate mutual information (slope -0.169 ± 0.011, intercept 0.302 ± 0.031), multivariate conditional mutual information (slope 0.181 ± 0.012, intercept -0.12 ± 0.037), entropy, mutual information of time-shifted environments, average pair-wise Pearson correlation, average Kullback-Leibler divergence. Information theoretic metrics were calculated for probability distributions (using 64 bins) of possible values of two signals *S_1 _*and *S_2 _*and a nutrients' signal. Nutrient's signals were taken either as a time delayed function of input signals *N *(see Figure 3), or as a shifted by -500 time steps nutrients' signal *N_shifted _*(i.e. with eliminated time delay relative to the input signals). Information theoretic metrics were calculated as: **MI**(*S_1_*; *S_2_*; *N*) = **H**(*S_1_*) + **H**(*S_2_*) + **H**(*N*) - **H**(*S_1_*;*S_2_*) - **H**(*S_1_*;*N*) - **H**(*S_2_*;*N*) + **H**(*S_1_*;*S_2_*;*N) *and **MI**(*S_1_*; *S_2_*|*N*) = **MI**(*S_1_*;*S_2_*) - **MI**(*S_1_*; *S_2_*; *N*); joint entropy is defined as $H\left(X_{1},...,X_{n}\right)=\sum_{x_{1}\in X_{1}\ldots }\sum_{s_{n}\in X_{n}}p\left(x_{1},...,x_{n}\right)\log\left(p\left(x_{1},...,x_{n}\right)\right)$ and mutual information is defined as $MI\left(S_{1};S_{2}\right)=\sum_{s_{1}\in S_{1}}\sum_{s_{2}\in S_{2}}p\left(s_{1},s_{2}\right)\log\left(\frac{p\left(s_{1},s_{2}\right)}{p_{1}\left(s_{1}\right)p_{2}\left(s_{2}\right)}\right)$, where (*p*(*x*_1_,...,*x_n_*) and *p_i _*(*x*) are joint and marginal probability distribution functions, respectively. Kullback-Leibler divergence is calculated as: **K-L Div**. $\left(S||N\right)=\sum_{i}p\left(S_{i}\right)\log\left(\frac{p\left(S_{i}\right)}{p\left(N_{i}\right)}\right)$ for probability distributions *S *and *N*.

**Figure 8:**
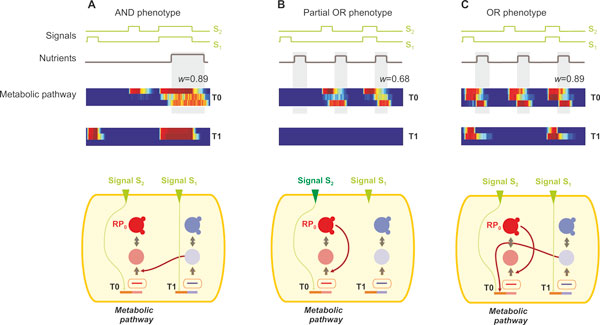
**Evolution during an AND-to-OR environmental transition**. Snapshots of the minimal network evolution for a single cell during an AND-to-OR transition are shown. T0 is the metabolic triplet, and RP0 is the response protein, whose expression is necessary for nutrient uptake (its expression profile is the third row in the heatmap). **(A) **Initially the cell is exposed to the AND environment, and exhibits a highly fit profile (Pearson correlation between RP0 and nutrients is 0.89). The minimal network illustrates the logic that has evolved: signal S1 activates the expression of gene and protein product of T1, which in turn catalyzes the translation of T0, while signal S1 should be presence for T0 gene expression, thus operating in an AND-like logic. **(B) **Upon exposure to the OR environment, the cellular network evolves to exhibit suboptimal phenotype, where only the second and third nutrient peaks are detected. **(C)** Final OR-like network evolves after several epochs from the intermediate network (shown in B). The cell expresses its metabolic protein during all nutrient occurrences.

The choice of environments is of paramount importance when it comes to changes in the rate of evolution. Contrary to what we expected, selecting an intermediate environment that strongly anti-correlates to the final environment, does not decelerate the evolutionary rate. The reason is that cell populations are mal-adapted to the second environment and the pre-evolved population is washed out within a few epochs during the transition, for the small population sizes of our simulations. We expect that this will also hold for larger population sizes, as the probability that a reversing mutation in the network arises is higher. Deceleration, however, can be achieved by first evolving the population at an environment of high complexity, which is not surprising. Interestingly, we observed deceleration in the case of weak positive or negative correlation between the intermediate and final environments. This is the case of evolution to the OR environment through an initial population that has pre-evolved in the AND environment. Although there is a weak correlation between the two environments (0.09), OR phenotypes do not arise until much later in the simulations (> 4000 epochs). Analysis of the evolutionary trajectory and population variation showed that this is a result of cells populating a local optimum in the fitness landscape, in full agreement with our initial hypothesis and Figure 1C. More specifically, within a few epochs after the transition to the OR environment (Figure 8A), the dominant phenotype in the population expressed the metabolic protein necessary to metabolize nutrients only during the second and third peak of nutrient occurrence (Figure 8B). However, due to the sub-optimal, but stable, hard-wiring of its regulatory network, the optimal OR phenotype only emerges 1000 epochs later.

## Conclusions

In this paper, we investigate the "guided evolution hypothesis", and provide evidence through *in silico *simulations that the rate of evolution can be both accelerated and decelerated by exposing a cell population to a series of environments. Towards identifying the rules that govern this phenomenon, we explored functions that can serve as a proxy for environmental complexity and similarity between environments. To understand how exposure to intermediate environments impacts evolutionary rates, we analyzed the fossil record of evolution on the networks and the population. Gathering and storing this amount of data would not been possible, without the development of a state-of-the-art, multi-scale, scalable microbial simulator that can run in HPC environments. Our results are insensitive to the particular evolutionary parameters, although simulations should be extended to more distinct environments in the future.

The implications of this work span many areas of biological research. First, we extend our current understanding of evolution by deriving a set of rules that explain the directional change of evolutionary rates when populations are exposed to a series of environments. By quantifying the environmental complexity, we made possible a more rigorous assessment of the evolutionary potential of microbial communities in complex environments. In the context of synthetic biology and bioengineering, our work may prove useful if the task is to control evolutionary rates without changing main environmental parameters (medium, mutagenic agents, and other abiotic parameters). The ability to guide evolution can be a powerful tool when it comes to fine-tuning of genetic constructs, designing principles for resilient biological circuits, and controllable biotechnological processes [14].

Our work can be extended to environments of higher dimensionality and complexity, since the scope of this study was limited to temporal signals that encode logical functions. As such, the metrics that we used to assess environmental complexity may not be applicable to environments that are spatially heterogeneous or are characterized by a multitude of parameters that affect bacterial physiology. In such cases, novel metric functions, which measure the difficulty of organisms to change and adapt when exposed to the new environment, should be constructed. In addition, our analysis, despite its novelty on merging models of gene regulatory and biochemical networks with population dynamics, did not exploit the possible link between the biological network organization and modular structure in a given environment, and the ability to generalize to new environments that may, or may not, be similar. Integration of emergent network characteristics and other environmental information has the potential to yield a more accurate depiction of the interplay between evolutionary rates and environmental organization.

Finally, it would be interesting to validate the guided evolution hypothesis in a laboratory setting. An obvious challenge is to characterize environmental similarity and complexity in static environments, as laboratory evolution experiments with shake flasks would be very difficult, if they were to reproduce a temporal correlation between the nutrients and environmental signals. However, chemostats have the capacity to couple nutrient availability to abiotic signals (temperature, pressure), and as such, they can provide a suitable platform to experimentally test this hypothesis.

## Competing interests

The authors declare that they have no competing interests.

## Authors' contributions

Corresponding author IT conceived the project; VM and IT designed the methods, analyzed the data, and wrote the manuscript. VM wrote the code and performed the experiments.

## References

[B1] Darwin C On the Origin of Species 1859 London: John Murray

[B2] Lee SH van der Werf JHJ Hayes BJ Goddard ME Visscher PM Predicting Unobserved Phenotypes for Complex Traits from Whole-Genome SNP Data Plos Genetics 2008 4 10 11 10.1371/journal.pgen.1000231 PMC2565502 18949033

[B3] Gardner A Zuidema W Is evolvability involved in the origin of modular variation? Evolution 2003 57 6 1448 1450 12894952 10.1111/j.0014-3820.2003.tb00352.x

[B4] Draghi J Wagner GR Evolution of evolvability in a developmental model Evolution 2008 62 2 301 315 10.1111/j.1558-5646.2007.00303.x 18031304

[B5] Ciliberti S Martin OC Wagner A Innovation and robustness in complex regulatory gene networks Proceedings of the National Academy of Sciences of the United States of America 2007 104 34 13591 13596 10.1073/pnas.0705396104 17690244 PMC1959426

[B6] Wagner A Robustness and evolvability: a paradox resolved Proceedings of the Royal Society B-Biological Sciences 2008 275 1630 91 100 10.1098/rspb.2007.1137 PMC2562401 17971325

[B7] Gerhart J Kirschner M The theory of facilitated variation Proceedings of the National Academy of Sciences of the United States of America 2007 104 8582 8589 10.1073/pnas.0701035104 17494755 PMC1876433

[B8] Kashtan N Noor E Alon U Varying environments can speed up evolution Proceedings of the National Academy of Sciences of the United States of America 2007 104 34 13711 13716 10.1073/pnas.0611630104 17698964 PMC1948871

[B9] Kashtan N Mayo AE Kalisky T Alon U An Analytically Solvable Model for Rapid Evolution of Modular Structure Plos Computational Biology 2009 5 4 14 10.1371/journal.pcbi.1000355 PMC2661363 19360090

[B10] Wilke CO Wang JL Ofria C Lenski RE Adami C Evolution of digital organisms at high mutation rates leads to survival of the flattest Nature 2001 412 6844 331 333 10.1038/35085569 11460163

[B11] Tagkopoulos I Liu YC Tavazoie S Predictive behavior within microbial genetic networks Science 2008 320 5881 1313 1317 10.1126/science.1154456 18467556 PMC2931280

[B12] Mozhayskiy V Miller R Ma K-L Tagkopoulos I A Scalable Multi-scale Framework for Parallel Simulation and Visualization of Microbial Evolution TeraGrid'11; Salt Lake Sity, UT 2011 doi:10.1145/2016741.2016749

[B13] Mozhayskiy V Tagkopoulos I In Silico Evolution of Multi-Scale Microbial Systems in the Presence of Mobile Genetic Elements and Horizontal Gene Transfer Lect Notes in Comput Sc 2011 6674 262 10.1007/978-3-642-21260-4_26

[B14] Porcar M Beyond directed evolution: Darwinian selection as a tool for synthetic biology Syst Synth Biol 2010 4 1 1 6 10.1007/s11693-009-9045-4 19821059 PMC2816224

